# Relaxation-Assisted Magnetization Transfer Phenomena for a Sensitivity-Enhanced 2D NMR

**DOI:** 10.1021/acs.analchem.3c03149

**Published:** 2023-11-27

**Authors:** Mihajlo Novakovic, Jihyun Kim, Xun-Cheng Su, E̅riks Kupče, Lucio Frydman

**Affiliations:** † Departments of Chemical and Biological Physics, Weizmann Institute of Science, Rehovot 7610001, Israel; ‡ State Key Laboratory of Elemento-organic Chemistry, College of Chemistry, Nankai University, Tianjin 300071, China; § Bruker Ltd., Banner Lane, Coventry CV4 9GH, United Kingdom; ∥ Department of Chemistry Education, Kyungpook National University, Daegu 41566, Republic of Korea

## Abstract

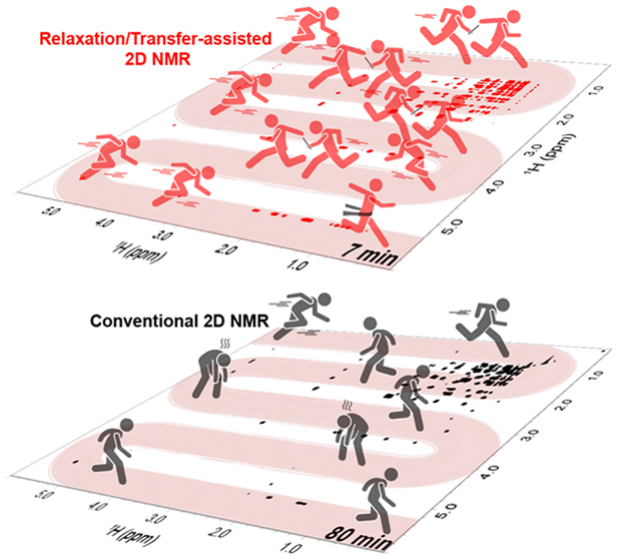

2D NOESY and TOCSY play central roles in contemporary NMR. We have recently discussed how solvent-driven exchanges can significantly enhance the sensitivity of such methods when attempting correlations between labile and nonlabile protons. This study explores two scenarios where similar sensitivity enhancements can be achieved in the absence of solvent exchange: the first one involves biomolecular paramagnetic systems, while the other involves small organic molecules in natural abundance. It is shown that, in both cases, the effects introduced by either differential paramagnetic shift and relaxation or by polarization sharing among networks of protons can provide a similar sensitivity boost, as previously discussed for solvent exchange. The origin and potential of the resulting enhancements are analyzed, and experiments that demonstrate them in protein and natural products are exemplified. Limitations and future improvements of these approaches are also briefly discussed.

## Introduction

Multidimensional NMR correlations play an important role in structural and dynamic studies of organic and biological molecules.^1−3^ These correlations are generated as a result of polarization transfers among spins, driven by stochastic processes like chemical exchange or the nuclear Overhauser effect (NOE),^4−7^ or through coherent homonuclear and heteronuclear J-couplings, as in TOtal Correlation SpectroscopY (TOCSY)^8,9^ and in Heteronuclear Single/Multiple Quantum Coherence (HSQC/HMQC) correlations,^10−12^ respectively. However, detecting these transfers, particularly homonuclear NOESY cross-peaks, is often challenged by the relatively low efficacy of the polarization transfer process. This limitation becomes even more critical when targeting cross-peaks involving labile or otherwise fast-relaxing protons. Intersite correlations in these instances are often lost by chemical exchange with the solvent or by self-relaxation, resulting in weak or no cross-peaks from such sites. We have recently discussed a number of avenues that actually exploit solvent exchange in order to enhance correlations involving labile protons.^13−18^ Using 2D NOESY as an example, the ensuing Looped-PROjected SpectroscopY (L-PROSY) approach proposes to replace the single *t*_1_ period, followed by the mixing cross-relaxation time typically involved in NOESY, by multiple, site-selective, *t*_1_-encoded Ramsey encodings of the peaks of interest, followed in each case by a cross-relaxation mixing time.^13,15^ During each such mixing time, the labile protons of interest will be repolarized through exchange processes with an unperturbed water ^1^H magnetization, acting as an efficient source for this remagnetization. As a result of this, a significant sensitivity enhancement can be imparted on the cross-peaks arising between the labile exchanging protons and the nonlabile sites, facilitating 2D NOESY (and, if driven by isotropic mixings, 2D TOCSY) correlations. Alternatively, one can target the labile peaks of interest one-by-one with frequency-selective irradiations, leading to selective magnetization transfer (SMT) experiments.^16^ Although lacking Fourier multiplexing, these experiments can also be designed to deliver NOE cross-peaks from labile sites with the added benefit of solvent repolarization enhancements. Further sensitivity improvements can be achieved by multiplexing these selective irradiation schemes with a Hadamard encoding,^19−21^ whereby SMT’s monochromatic frequency-selective pulses are replaced by polychromatic saturation or polychromatic inversion pulse blocks, perturbing multiple labile peaks in what will become the *F*_1_ frequency domain. By replacing the *t*_1_ time-domain encoding of L-PROSY with such Hadamard Magnetization Transfer (HMT) frequency-domain encoding, the efficiency of the 2D NMR measurement on a sparse, labile proton system can be increased even further, while still benefiting from the aforementioned exchange-driven solvent repolarization effects.

All of these methods have in common the reinstatement of the usual 2D NOESY/TOCSY sensitivity in correlations involving labile sites, despite the decoherence and losses imparted on these protons through solvent exchange. However, it is not the chemical exchange with the solvent *per se* that provides L-PROSY, SMT or HMT with their sizable sensitivity enhancements. Rather, it is the fact that, by leaving the solvent reservoir untouched, the labile and nonlabile ^1^H effective *T*_1_s can become very different: exchanges with the solvent will rapidly replenish the former’s polarization, while the latter will not benefit from this. As a result, the transfer from the labile to the nonlabile spins can be enhanced by looping the transfers: each time that the labile ^1^H has been *t*_1_-encoded or affected by a frequency selective saturation/inversion, repolarization from the solvent will allow its dialogue with the nonlabile sites to be renewed, for as long as the latter’s memory time allows. This means that if dealing with systems that naturally have very different *T*_1_ relaxation times, or if somehow the apparent *T*_1_ times of sites to be correlated can be made effectively very different, one could still exploit similar concepts in NMR settings that do not necessarily involve labile ^1^H sites exchanging with the solvent. The present study presents two examples where such effects are demonstrated and brought to bear for enhancing sensitivity, even in the absence of chemical exchange.

## Experimental Methods

### Sample Preparation

The sequence for the human Ubiquitin E64C mutant was prepared in a pET28a vector. *E. coli* BL21 (DE3) Rosetta was transformed for protein overexpression. Grown in 2 L of LB medium at 37 °C until mid log phase, the bacterial cells were induced by the addition of 0.5 mM IPTG for protein expression overnight at 15 °C. The bacterial cells were collected by centrifugation and then lysed by sonication. The target proteins were purified by a DEAE column (DEAE Sepharose FF, GE Healthcare Biosciences), followed by a size exclusion column (HiLoad 16/600 Superdex 75, GE Healthcare Biosciences). Pure ubiquitin fractions were determined by SDS-PAGE. Labeling with BrPSPy-DO3MA-Tm(III) was performed as described earlier.^47^ Protein samples were initially dissolved in 20 mM MES buffer at pH 6.4 at 1 mM concentration and then exchanged to a pH 6.4 NMR buffer consisting of 20 mM phosphate and 50 mM NaCl.

Menthol and nonadecanoic acid (SigmaAldrich) were prepared at 100 mM concentration, and cholesterol was prepared at 50 mM in acetone-*d*_6_ (Merck).

### NMR Spectroscopy

NMR experiments were conducted using either a 14.1 T Bruker magnet equipped with an Avance III console and TCI Prodigy probe or a 1 GHz, 23.5 T Bruker Avance Neo instrument equipped with a TCI cryoprobe and processed using Bruker TopSpin software. L-PROSY HSQC-TOCSY data were collected using an 11.7 T Magnex magnet (Abingdon, U.K.) run by a Varian iNova console (Palo Alto, U.S.A.) and equipped with a triple resonance HCN Varian 5 mm probe possessing *x*, *y*, and *z* gradients. These data were processed and integrated by using MestreNova software (version 11.0.4, Mestrelabs Research, Spain). Experiments with paramagnetically labeled Ubiquitin are acquired at 303 K and with small molecules at 298 K. Sixteen scans per-increment were used in the HMT experiments on the paramagnetically labeled Ubiquitin, and 8 scans per-increment in experiments on the small molecules. In all cases, the recycling delay *d*_1_ was 2 s. Conventional NOESY and TOCSY experiments were acquired with standard Bruker pulse sequences. HMT and SMT pulse sequences were written in-house and are available for download in either our group’s Web site https://www.weizmann.ac.il/chembiophys/Frydman_group/software or in the Bruker user library https://www.bruker.com/protected/en/services/bruker-user-library/liquids.html. The HMT-shaped pulses used in the paramagnetic Ubiquitin experiments were sinc180 pulses with bandwidths of 40 Hz, automatically generated on-the-fly by WaveMaker. Similar sinc180 pulses were used for experiments with small molecules. As SMT experiment allows different pulse bandwidths specific to the sites that are being probed,^16^ we used bandwidths from 15 to 40 Hz depending on the available spectral resolution (15 Hz in crowded regions and 40 Hz for isolated peaks). A DIPSI2 isotropic mixing of 5.6 kHz (corresponding to the p90 of 45 μs) was used. The detailed description of how to set up and apply HMT and SMT experiments is available in the Bruker user library.

### NOESY Cross-Peak Enhancements in Paramagnetically Labeled Proteins

A natural instance where the conditions described in the preceding paragraph could be met, arises in systems where sites have short *T*_1_s because of their proximity to paramagnetic centers.^22,23^ This arises for instance in paramagnetic proteins, where fast-relaxing sites proximate to the paramagnetic center are notable because of their inability to yield significant NOE or TOCSY cross-peaks. However, their transfer to slower-relaxing sites could be enhanced using approaches similar to those introduced above, despite the absence of a constant water repolarization. In this case, it would be rapid paramagnetic relaxation that serves as the repolarization engine, resupplying fresh magnetization and thereby enhancing the transfer efficiency of NOESY and TOCSY experiments if probed by L-PROSY or MT-based procedures.

**Figure 1:**
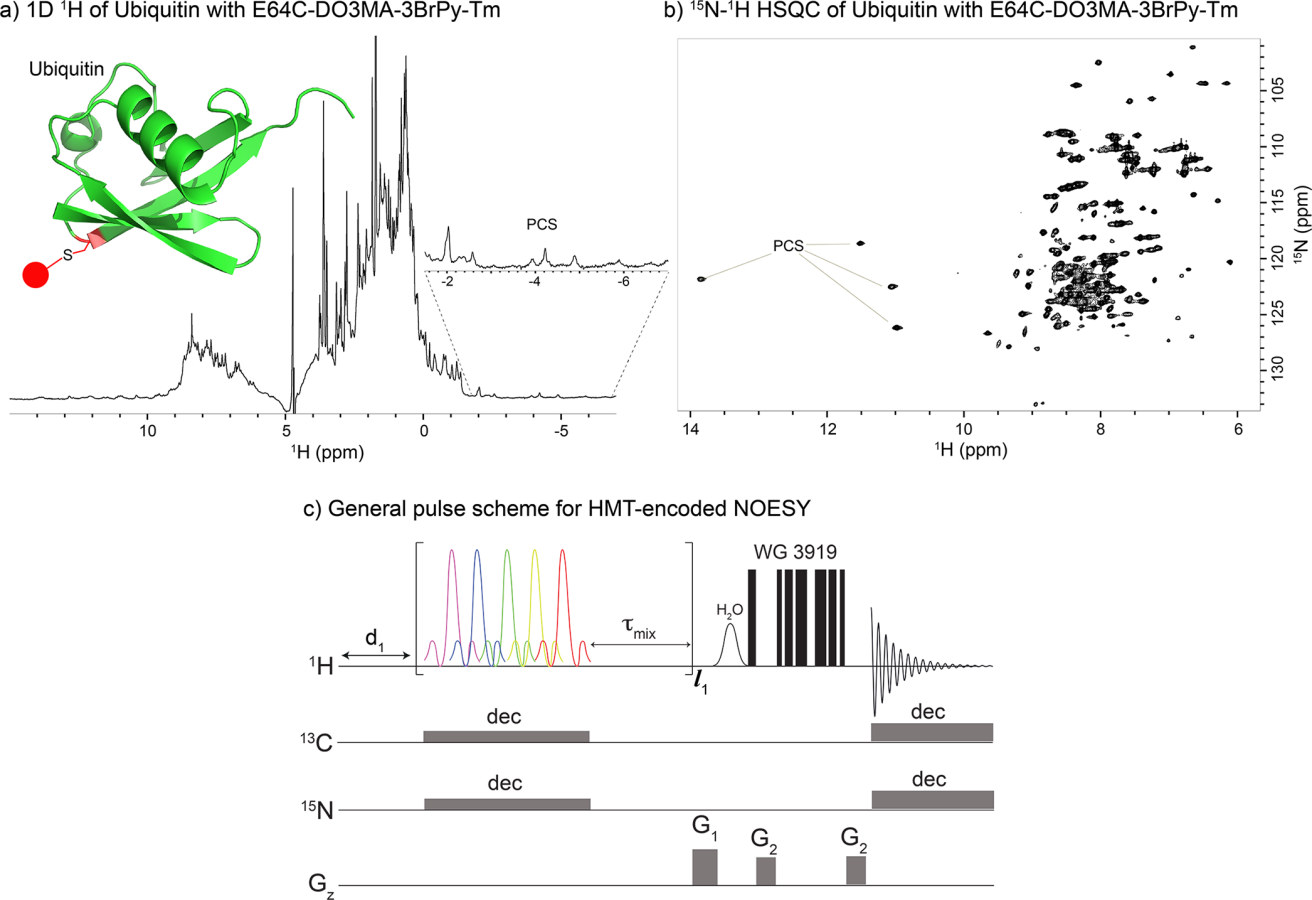
(a) 1D proton spectrum of E64C Ubiquitin protein tagged with Thullium-based spin label DO3MA-3BrPy, shown as a red dot in the structure. Note that multiple resonances experience large pseudocontact shifts (PCS). (b) ^15^N–^1^H HSQC 2D spectrum some of the Tm-tagged ubiquitin, showing some of the amide resonances exhibiting large PCSs. These shifts facilitate the targeting of these resonances by a selective pulses. (c) General HMT-encoded NOESY sequence (with optional decoupling for labeled samples) used to enhance correlations in this system. As this Ubiquitin sample was solely ^15^N labeled, only ^15^N decoupling was used in this study.

In order to test the enhancements achievable in these scenarios, we examined a paramagnetic ubiquitin sample containing a thulium-based spin label: E64C-DO3MA(R)-3BrPy-Tm(III). This is a ubiquitin molecule incorporating a thiol-bound DOTA-derivative, (2*R*,2′*R*,2″*R*)-2,2′,2″-(10-(5-bromo-4-(phenylsulfonyl)pyridin-2-yl)methyl)-1,4,7,10-tetraazacyclododecane-1,4,7-triyl)tripropanoic acid (DO3MA-3BrPy), in a complex with Tm(III).^24^ All the sites that in this Ubiquitin are in close proximity to the spin label experience large pseudocontact shifts (PCS),^25^ that significantly displace them from the rest of the protein ^1^H signals. This is clearly observable from −7 to −2 ppm for methyl protons and from 10 to 15 ppm proton regions for amides (Figure 1a). These sites also exhibit a fast, paramagnetically induced relaxation that complicates homonuclear polarization transfer correlations originating in these ^1^Hs. As a consequence of this and of the experiment’s intrinsically low SNR, NOESY acquisitions are not regularly performed when studying such paramagnetically labeled proteins. The alternatives introduced before can help to overcome these obstacles, yielding NOE correlations involving these fast-relaxing species, and yielding structural information about the region proximate to the paramagnetic spin label. Such enhancements could be obtained using the *t*_1_-encoded L-PROSY technique; still, we decided here to focus on data acquired using the HMT scheme (Figure 1b), which is aided in this case by the fact that one can then solely concentrate on the few and well resolved peaks exhibiting the PCS. HMT encoding pulses could be tuned easily in these systems, and thanks to their compressed-sensing features, the overall experiments could be acquired in a fraction of the time of their L-PROSY counterpart.

**Figure 2:**
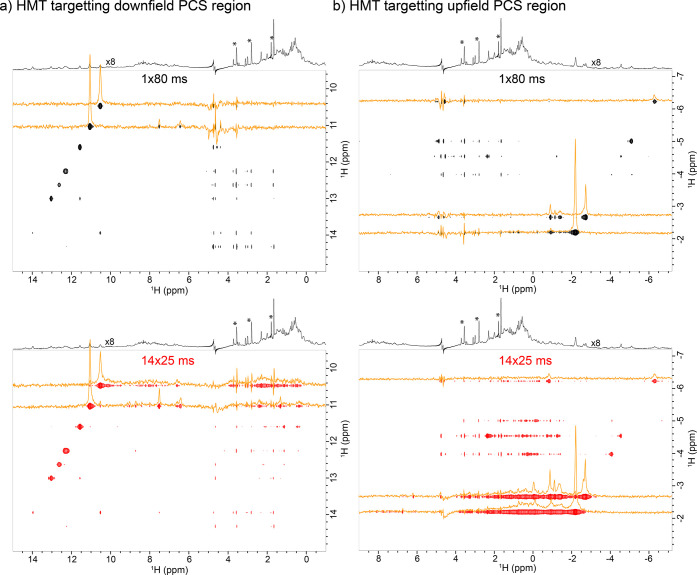
Assessing the effects of looping a Hadamard encoded HMT-NOESY experiment when targeting Ubiquitin’s paramagnetic resonances: (a) Experiencing downfield PCSs; (b) Idem but for upfield PCSs. Mixing time for the single-loop experiment was chosen 80 ms to accommodate fast relaxation, while the HMT experiment was performed with 14 loops and 25 ms per loop. The slices extracted from the spectra (orange traces) demonstrate the substantial gains achievable by looping of the inversions for illustrative resonances. The single loop HMT experiment took around 9.5 min to acquire, while the looped experiment was only 1 min longer. Shown on top of each 2D spectrum is an external WATERGATE 3919 1D spectrum to better visualize the spectrum of this paramagnetically labeled Ubiquitin (regions containing PCS residues are enlarged 8× to be visible in the projection). MES buffer peaks are labeled with asterisk.

Figure 2 demonstrates the enhancements that these principles can generate by comparing a singly encoded HMT NOESY experiment (top row) with a looped HMT counterpart. Both experiments concentrated on PCS-affected proton signals. Fast relaxation of the targeted sites sets up an upper boundary for the NOESY mixing time of ca. 80 ms period, but this setting originates only few, relatively weak cross-peaks. By contrast, the looped HMT experiment builds up NOE effects over 350 ms (Figure 2, bottom row), providing the substantial cross-peaks gains evidenced by the highlighted (orange) cross sections. These enhancements range between 3- and 8-fold, for the upfield- and downfield-shifted peaks, respectively.

### Cross-Peak Enhancements in Small Molecules Aided by J-Driven Repolarizations

**Figure 3:**
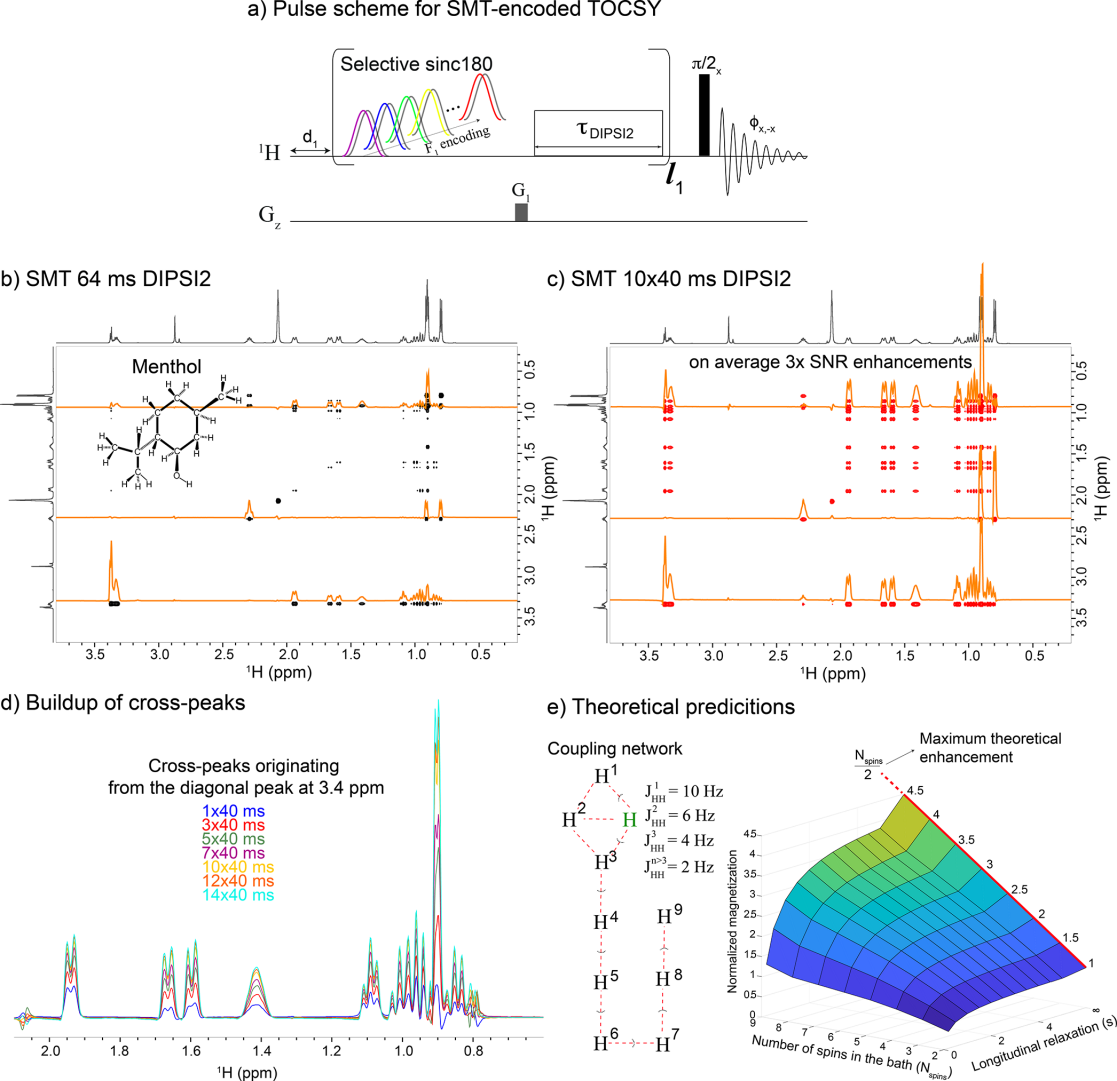
(a) Pulse sequence for SMT encoding of TOCSY correlations in small molecules. After picking peak frequencies from a standard 1D spectrum, an SMT experiment looping selective sinc180 pulses followed by DIPSI2 mixing periods is used to encode sequentially chosen resonances in F1, utilizing sensitivity enhancements and providing 2D TOCSY spectra. Off-resonance reference scan is used for phase-cycling as explained for the SMT experiment.^16^ (b, c) Comparison of sequentially encoded TOCSY experiments on 100 mM menthol using conventionally optimal 64 ms DIPSI2 mixing and a SMT-like experiment utilizing 10 loops of 40 ms DIPSI2 mixing. While the acquisition of the looped SMT lasted ca. 15% longer than the nonlooped experiment, the projections (highlighted in orange) extracted from several chemical shifts in the spectra show average 3× SNR enhancements vs TOCSY cross-peaks without looping. The ^1^H spectrum of menthol is shown as F1 and F2 external projections. (d) Buildup of structurally relevant signals visualized by following cross-peaks stemming from the proton resonance at 3.4 ppm upon looping. (e) Simulation based on a Bloch-McConnell model including up to 9-spins in the pool. The figure shows that the enhancements achievable by this method depend on the longitudinal relaxation of the receiving spin pool (memory time of the receiving spins sets up the steady-state of the SMT buildup, equivalent to *T*_1ρ_ in the case of an isotropic mixing) and the total number of spins in the receiving spin pool. While this model assumes a stochastic exchange and TOCSY is a coherent process, a sufficiently large distribution of *J*-couplings is hypothesized to redistribute the polarization similarly.

**Figure 4:**
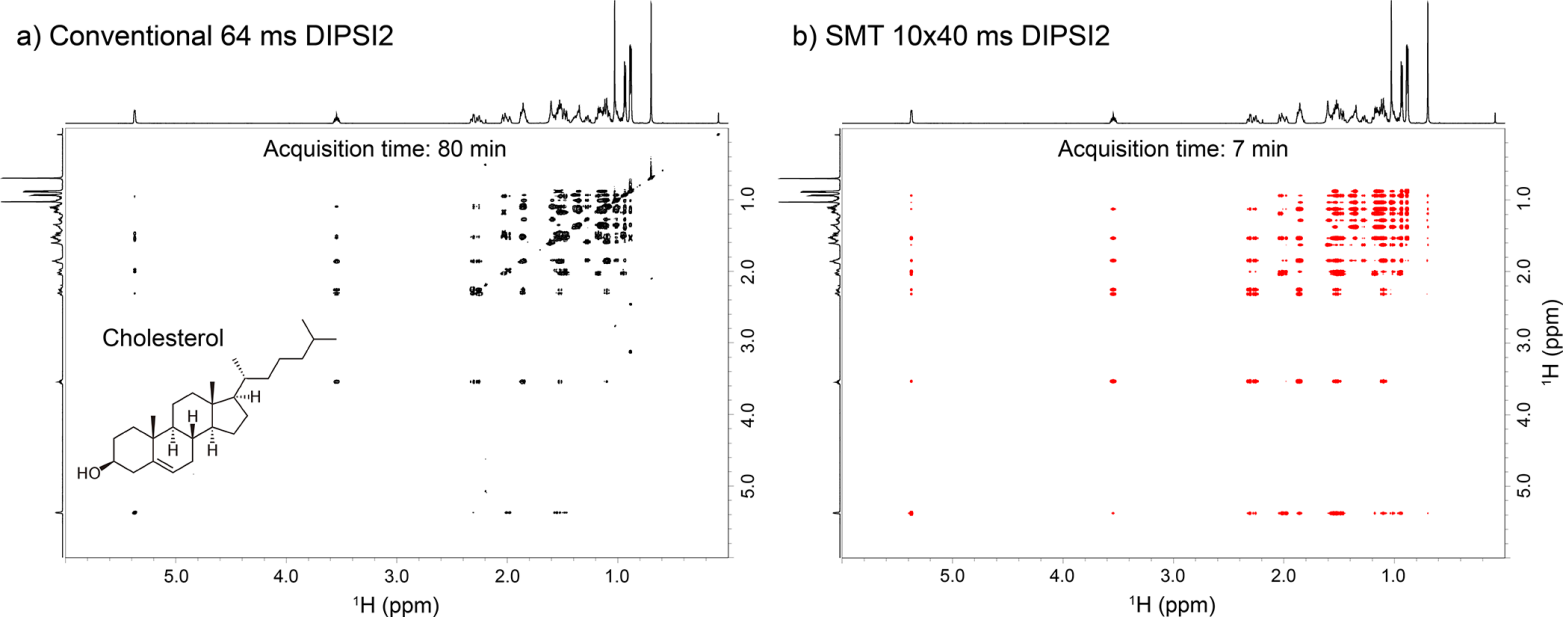
(a) Conventional TOCSY spectrum acquired using 64 ms DIPSI2 isotropic mixing. (b) SMT counterpart acquired using 10 loops and 40 ms mixing per loop on a 50 mM cholesterol sample. ^1^H 1D spectra of cholesterol are shown as F1/F2 external projections.

Another possibility for reducing the effective *T*_1_ relaxation times and thereby enhancing NOESY/TOCSY correlations by the aforementioned strategies arises in small molecules containing a large network of J-coupled protons. In such cases one can conceivably repolarize selectively targeted species–for instance, sites encoded in HMT or SMT-type experiments, via so-called ASAP (Acceleration by Sharing Adjacent Polarization) isotropic mixing processes spreading “underused” polarization, throughout the spin-coupled network.^26−30^ Even though achieving similar end results as water-driven repolarization experiments relying on chemical exchange, the “*T*_1_ shortening” mechanisms acting in these J-driven repolarizations would be rather different.^26,29^ In particular, if considering how to raise the efficiency for TOCSY-based transfers, one could use the isotropic mixing block that provides the total correlation 2D spectroscopy information and also to repolarize the perturbed spins being addressed. Figure 3a illustrates an SMT experiment built on such basis that can be used to sequentially encode TOCSY on specific hydrogens in a small molecule. In this particular realization, resonances are selectively perturbed with a monochromatic inversion pulse, and this encoding is followed by a DIPSI2 mixing where correlations are made. Concurrently, the inverted spin(s) will recover fast, thanks to receiving polarization from the now DIPSI2-recoupled, J-interconnected spin pool. Repeating the inversion/DIPSI2 block multiple times thus enhances the final cross-peaks, for reasons akin to those helping in solvent-enhanced L-PROSY/HMT/SMT methods. This is illustrated in Figure 3b and c, which compare SMT-encoded TOCSY cross sections recorded on three menthol sites using the 64 ms duration that would be used in conventional experiments, against a 40 ms mixing block that was repeated 10 times (40 ms of TOCSY being enough to repolarize these protons to ∼90% of thermal magnetization, see Figure 3 in ref (29)). Large sensitivity gains are evidenced by the latter’s TOCSY cross-peaks, as can be appreciated by comparing projections extracted from several *F*_1_ frequencies in the spectra. Figure 3d illustrates the buildup of cross-peaks upon looping; notice how 40 ms leads to a nearly complete repolarization of the targeted proton, enabling gains to persist even after 14 loops of repeating the same mixing period. As shown by the simulation results in Figure 3e, the buildup of the cross-peaks arising in this kind of experiments, and hence the achievable enhancements upon looping, will be highly dependent on the number of spins available in the overall reservoir and on their relaxation properties. In this sense, and although addressing smaller pools of protons at any given time, SMT-based schemes may benefit further from these polarization-sharing strategies than broadbanded HMT-like schemes. Similar enhancements were obtained for cholesterol as follows from the comparison between conventional TOCSY and SMT experiments shown in Figure 4. Notice the high quality, full total correlation that in the latter case could be obtained in under 7 min, compared to a conventional acquisition that took ∼12 times longer to acquire.

**Figure 5:**
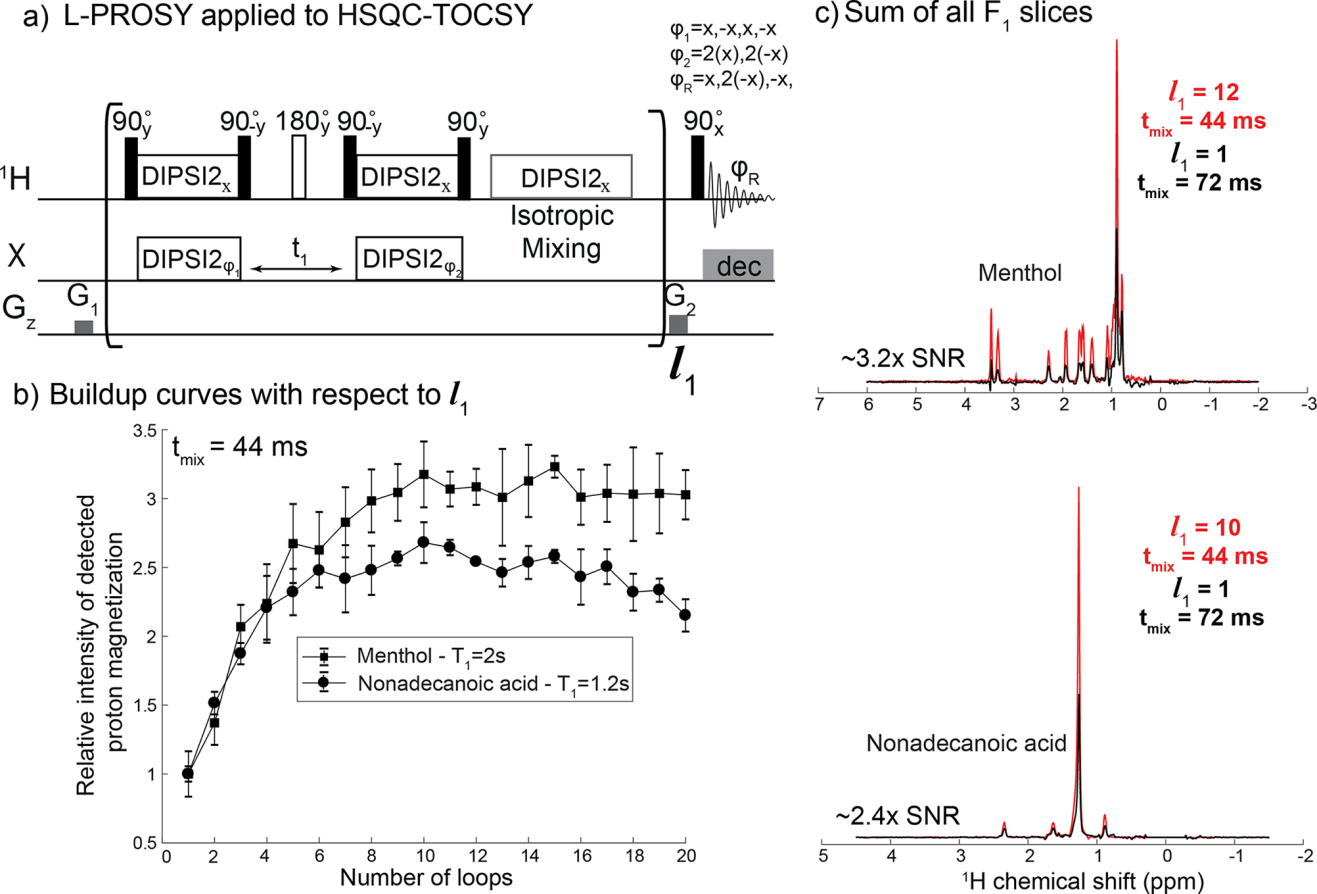
(a) 2D L-PROSY HSQC-TOCSY pulse sequence designed for a sensitivity-enhanced detection of the TOCSY correlations. (b) Buildup curves of the ^13^C amplitude modulation with respect to the number of loops *l_1_* of sequence (a), acquired for menthol and nonadecanoic acid. (c) Comparisons of the sum of *F*_1_ traces from conventional and from L-PROSY HSQC-TOCSY experiments, demonstrating the per-scan enhancements for the total ^13^C-selected resonances. Data were collected on a Varian iNova console (Palo Alto, U.S.A.) and equipped with a triple resonance HCN Varian 5 mm probe.

The substantial SNR gains presented above arise in a particularly convenient form when attempting to enhance heteronuclear correlations of low-abundance species, including HSQC-TOCSY experiments run on compounds at natural abundance.^31^ 2D HSQC-TOCSY is a widely used experiment to establish correlations between heteronuclei such as ^13^C or ^15^N, and all protons partaking in a spin coupling network involving the heteronuclei-bonded ^1^H.^31^ It follows that a majority of protons in these experiments, which are those bonded to ^12^C or ^14^N, are passive spectators in natural abundance compounds. The use of short homonuclear Hartmann–Hahn isotropic mixing periods gives these inactive protons the possibility to repolarize the ^13^C-bound species, transferring their equilibrium magnetizations through homonuclear scalar couplings, and reducing the effective relaxation time of an expended, ^13^C-bound proton, down to ≈0.1 s. This ASAP principle^26,27^ was combined in Figure 5 with the L-PROSY idea, to impart into HSQC-TOCSY experiments the kind of sensitivity gains addressed in the preceding section for small molecules–only that, instead of relying on selective chemical shift excitations, by relying on selective isotopomer excitations.

A number of ways to implement these looped experiments were assayed; it was found that the most practical way to extend ASAP/L-PROSY ideas to HSQC-TOCSY correlations was by incorporating them into cross-polarization (CP) transfer schemes, using composite isotropic sequences as part of both the hetero- and the homonuclear transfers mixing stages. This led to the scheme illustrated in Figure 5a, involving the looping of a *t*_1_-block that passes magnetization between the ^1^H reservoir and the ^13^C state, while always bringing the former back into the longitudinal, *z*-state. At the same time, however, the extensive use of isotropic mixing periods in each loop of the sequence serves to amplitude-modulate the full ^1^H network connected by mutual *J*_HH_ couplings, with the ^13^C frequencies encoded over *t*_1_. Notice that, in contrast to a conventional 2D HSQC-TOCSY experiment where all the polarization that is eventually detected initiates from the ^13^C-bound protons, this L-PROSY scheme repeatedly shares the ^13^C modulation with the full pool of *J*_HH_-coupled protons, which in turn repolarizes back in every block the ^13^C-coupled proton from which the t_1_ evolution originates. In such sense, this experiment is reminiscent of the Heteronuclear Frequency Label EXchange (HetFLEX) experiment proposed to detect heteronuclear spectra using solvent-driven chemical exchange,^33,34^ and to the Fourier-Encoded Saturation Transfer (FEST) experiment relying on ^1^H–^1^H spin diffusion to achieve a similar effect in solids.^35^ After multiple such repetitions, the modulation imparted by the ^13^C on the full proton spin pool becomes significantly enhanced. Figure 5b,c exemplifies this with results obtained on natural-abundance menthol and nonadecanoic acid. As was the case for the experiment introduced in Figure 3, the precise extent of the sensitivity improvement will also in this case depend on multiple parameters, including the number of protons in the spin-coupled networks of the molecule, their *J*-couplings, and the proton *T*_1_ relaxation dictating the maximum plateau that can be reached by the process. For the cases here studied, ≥300% signal enhancements vis-à-vis conventional experiments were observed.

## Discussion and Conclusions

This study explored two novel applications of repolarizing principles to refresh the magnetization and thus enhance the sensitivity of looped TOCSY- and NOESY-based experiments in systems that do not undergo exchanges with an abundant solvent. Instead of repolarizing by chemical exchange, the targeted spin pool was assumed to either naturally have much shorter relaxation times than the recipient protons being correlated or to be amenable to pulse sequence manipulations that can effectively endow it with significantly higher relaxation rates. As an example of a fast-relaxing pool in which sensitivity-enhanced 2D correlation NMR spectra could be obtained, we focused on a paramagnetic molecule. In particular, a Tm(III)-labeled Ubiquitin provided both the chemical shift dispersion facilitating the kind of selective excitation needed by these experiments and the shorter relaxation times that these also demand. Sizable enhancements of up to 8× could be observed in NOE-driven correlations in these systems by the use of polychromatic, Hadamard-encoded manipulations.

In a different context, small molecules could also be repolarized without help from chemical exchange with the solvent via isotropic sequences sharing the ^1^H polarization along a fairly extensive proton network. Two scenarios were then investigated: one focused on 2D homonuclear acquisitions; the other involving an additional low-abundance heteronucleus. Spectral overcrowding in the first of these cases made the encoding better suited for selective frequency-domain irradiations rather than *t*_1_-based Fourier schemes; by contrast, the latter was found easier when involving a heteronuclear evolution. Still, as in the HetFLEX and FEST cases, a limitation of the resulting heteronuclear-encoding method is its sensitivity to *t*_1_ noise artifacts.^35,36^ This results from the inherent demand of these sequences to employ hard pulses throughout their course to preserve the full pool of ^1^H as intact as possible and then detect on the latter the magnified effects of the heteronuclei modulation. Thus, even though the ^1^H–^1^H polarization sharing scheme in Figure 5 may lead to HSQC-driven modulations of up to 4% of the total ^1^H magnetization (1% involving the ^13^C-bound protons and another ≈3–4% the ^12^C-bound proton network), one still relies only on the transmitter and receiver accuracies to phase-cycle out the remaining, unmodulated proton signals. Any pulse and phase imperfections throughout the looping process will then lead to multiplicative *t*_1_ noise artifacts in the final, observed bulk ^1^H signal along the *F*_1_ dimension. We have seen this kind of limitations as F1-like noise in the small molecule spectra (Supporting Information, Figure S1); solutions to this problem based on postprocessing, reference deconvolutions using external standards and on compressed sensing, are being considered.^37−41^ Another possible application of these ideas is to enhance the sensitivity of selective ROESY experiments. These extensions are also being investigated.

The present work extended principles that were demonstrated in both solvent-exchanging and solid-state systems to correlations between fast- and slow-relaxing reservoirs in paramagnetic and small molecules. Yet an additional scenario can be visualized for these effects, whereby small molecules binding and then rapidly releasing from large ones, will also benefit from similar relaxation enhancements.^42^ To a certain extent, dark-state exchange saturation transfer (DEST)^43^ and *in vivo* magnetization transfer (MT) experiments benefit from these features, by exploiting the different resonance offsets and different relaxation characteristics (including different T2) presented by species exchanging between solids/semisolids, and bulk solutions.^44−46^ It is to be expected that other instances where spectrally distinct, selectively addressable binding processes will allow one to exploit HMT/SMT principles to enhance structurally relevant information will also arise.

## Supporting Information Available

Comparison between nonlooped and looped 2D L-PROSY HSQC-TOCSY experiments (PDF)
