# Supplementary material for: Relaxation-Assisted Magnetization Transfer Phenomena for a Sensitivity-Enhanced 2D NMR
Source: Anal Chem. 2023 Nov 27;95(49):18091–8. doi: 10.1021/acs.analchem.3c03149 (PMC10719887; doi:10.1021/acs.analchem.3c03149)
Supplement: Supplementary file 1 — ac3c03149_si_001.pdf [file ac3c03149_si_001.pdf]

Supporting Information for

Relaxation-assisted magnetization transfer phenomena for sensitivity-enhanced 2D NMR

Mihajlo Novakovic[1,2], Jihyun Kim[1], Xun-Cheng Su[3], Ēriks Kupče[4], Lucio Frydman[1,*]

[1]Departments of Chemical and Biological Physics, Weizmann Institute of Science, Rehovot 7610001, Israel
[2]Current address: Institute of Biochemistry, Department of Biology, ETH Zürich, Hönggerbergring 64, 8093 Zürich, Switzerland
[3]State Key Laboratory of Elemento-organic Chemistry, College of Chemistry, Nankai University, Tianjin 300071, China
[4]Bruker Ltd, Banner Lane, Coventry CV4 9GH, UK
*Email: lucio.frydman@weizmann.ac.il

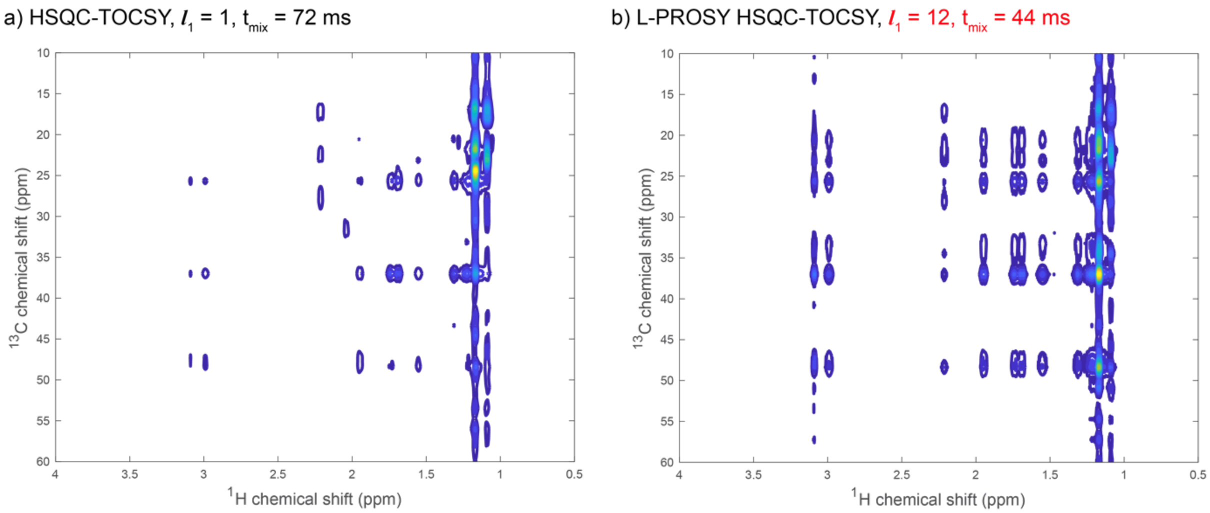

Figure S1. Comparison between a) non-looped and b) looped 2D L-PROSY HSQC-TOCSY experiment acquired on a menthol sample illustrating the enhancements as presented in Figure 5c, as well as extensive t_1 noise ridges along the spectrum's F1 dimension (right). This results from the inherent demand of these sequences to employ hard pulses throughout their course to preserve the full pool of ^{1}Hs as intact as possible, and then detect on the latter the magnified effects of the heteronuclei's modulation.
